# Airway coach project: development of a machine learning–based model using clinical and ultrasound parameters to support videolaryngoscopy strategy

**DOI:** 10.1186/s12871-026-03943-4

**Published:** 2026-06-18

**Authors:** Miguel Angel Fernández-Vaquero, Ricardo Oyarzun-Silva, Pablo Hernández-Hernández, Manuel Á. Gómez-Ríos, Cristina Petrisor, Stefano Falcetta, Sara H. Gomes, Nekari De Luis-Cabezón

**Affiliations:** 1 Department of Anaesthesiology and Critical Care, Clínica Universidad de Navarra, Madrid, Spain; 2 Department of Radiation-Oncology Puerta, Mar University Hospital, Cadiz, Spain; 3 Department of Anaesthesiology, Puerto Real University Hospital, Cadiz, Spain; 4 Anaesthesiology and Perioperative Medicine, Complejo Hospitalario Universitario de A Coruña, A Coruña, España; 5 Department of Anaesthesia and Intensive Care II, “Iuliu Hațieganu” University of Medicine and Pharmacy Cluj-Napoca, Clinical Emergency County Hospital of Cluj, Cluj-Napoca, Romania; 6 Clinic of Anaesthesiology and General Intensive Care, Respiratory and Major Trauma, AOU delle Marche Ancona, Ancona, Italy; 7 School of Medicine, Life and Health Sciences Research Institute (ICVS), University of Minho, Hospital CUF, Porto, Portugal; 8 Department of Anaesthesiology and Critical Care, Basurto University Hospital, Bilbao, Spain

**Keywords:** Airway management, Artificial intelligence, Clinical decision support systems, Machine learning, Tracheal intubation, Ultrasonography, and Video-assisted techniques and procedures

## Abstract

**Background:**

Videolaryngoscopy (VL) is recommended as a first-line technique for tracheal intubation; however, existing airway assessment tools—largely derived from direct laryngoscopy—provide limited guidance for videolaryngoscopy-specific decisions such as blade selection or anticipated adjunct use. Point-of-care airway ultrasound offers additional anatomical information that may complement conventional clinical assessment. Multimodal approaches integrating clinical and ultrasound-derived variables may improve pre-procedural videolaryngoscopy planning.

**Methods:**

In this single-centre prospective observational study, 250 adults (ASA physical status I–III) undergoing elective surgery were assessed preoperatively using clinical variables and point-of-care airway ultrasound. Videolaryngoscopic intubation was initiated with a Macintosh-type blade and prospectively classified according to procedural performance: Grade 0 (Macintosh blade without adjuncts), Grade 1 (Macintosh blade requiring adjuncts), and Grade 2 (need to switch to a hyperangulated device). A supervised machine-learning approach was used to develop a multiclass classification model integrating clinical and ultrasound-derived variables, using stratified cross-validation on a training dataset and evaluation on an independent test set.

**Results:**

In the independent test set, a gradient boosting model demonstrated good discriminative performance for videolaryngoscopy strategy classification (AUC 0.95; accuracy 92%). Performance varied across outcome categories, with lower F1-scores in the less frequent classes (F1-score 0.75 for Grade 1 and 0.67 for Grade 2). Classification was driven by a combination of tongue-related ultrasound parameters, anterior neck soft-tissue distances, body-mass index, and age.

**Conclusions:**

In this single-centre observational study, a machine learning–based model integrating clinical and airway ultrasound variables was developed to support videolaryngoscopy strategy planning. By targeting a functional, videolaryngoscopy-specific outcome that reflects procedural performance rather than glottic view alone, this multimodal, data-driven approach demonstrates feasibility and warrants further evaluation in independent populations before clinical implementation.

**Trial registration:**

This study was registered at Clinical Trial.gov NCT 06925009.

**Supplementary Information:**

The online version contains supplementary material available at 10.1186/s12871-026-03943-4.

## Introduction

Tracheal intubation (TI) is a cornerstone of anaesthesia and critical care practice. More than 320 million surgical procedures are performed worldwide each year, and up to 40% of intensive care unit admissions require airway instrumentation [1]. Despite advances in airway management, difficult TI (5–8%) and failed TI (0.05–0.35%) remain important contributors to morbidity, mortality, and medicolegal risk [2]. Conventional bedside airway assessments, including the Mallampati score, thyromental distance, and interincisor gap, have demonstrated limited discriminative ability, highlighting the need for more informative and objective approaches [3].

Point-of-care airway ultrasound (POCUS) provides a non-invasive and reproducible assessment of upper airway soft-tissue anatomy and has been proposed as a complementary tool to traditional clinical evaluation [4]. In parallel, the widespread adoption of videolaryngoscopy (VL) has transformed airway management by improving laryngeal visualization and first-pass success, and it is now recommended as a first-line technique for tracheal intubation in multiple international guidelines [5–10]. However, improved glottic visualization alone does not necessarily translate into successful tracheal tube delivery. Most existing prediction models remain extrapolated from direct laryngoscopy (DL) paradigms and provide limited guidance for videolaryngoscopy-specific decisions, such as blade selection or the anticipated need for adjuncts.

Importantly, videolaryngoscopy has modified the relationship between glottic visualization and successful tracheal tube delivery. In contemporary practice, an adequate or even excellent glottic view may coexist with difficulty advancing the tracheal tube—a phenomenon often described as “can see, can’t intubate.” Large contemporary series of universal videolaryngoscope use have confirmed that intubation difficulty may still occur despite optimal visualization, particularly when adjuncts or changes in blade geometry are required [11]. This observation underscores the limitations of visual grading systems alone and highlights the importance of functional, performance-based assessment of videolaryngoscopy.

Videolaryngoscopes are not a homogeneous group of devices. Macintosh-type videolaryngoscope blades preserve familiar geometry and allow both direct and indirect laryngoscopy, whereas hyperangulated blades are designed to optimize glottic exposure in anatomically challenging airways. Experimental and clinical evidence indicates that blade geometry substantially influences tracheal tube trajectory and delivery mechanics, even in the presence of an excellent laryngeal view [12]. Although hyperangulated videolaryngoscopes are often considered in patients with known or anticipated difficult airways, their use typically requires tracheal tube introducers or stylets. Adjuncts such as stylets and bougies are therefore integral components of the videolaryngoscopic intubation process, and recent evidence suggests that their use—particularly rigid stylets—may be associated with airway trauma in certain clinical contexts [13, 14]. Anticipating the need for adjuncts and identifying when escalation to a different blade geometry is likely may therefore be clinically relevant when planning a videolaryngoscopy strategy.

Machine learning (ML) provides a methodological framework for integrating heterogeneous clinical, demographic, and imaging-derived data. ML approaches have shown promise in perioperative risk stratification and airway assessment [15, 16]. Integrating clinical characteristics, demographic data, and point-of-care ultrasound measurements within a machine-learning framework may help characterize videolaryngoscopy-related procedural difficulty and support pre-procedural planning. In this context, we designed a prospective observational study to develop and internally validate a supervised machine-learning model for multiclass classification of videolaryngoscopy-related procedural difficulty.

The objective of this study was to develop a multimodal machine learning–based model, referred to as the Airway Coach Project, to classify videolaryngoscopy-related procedural difficulty using a supervised multiclass machine-learning approach integrating clinical and airway ultrasound variables, with internal validation through cross-validation and an independent test set.

## Methods

### Study design and oversight

We conducted a single-centre, prospective observational study, which was approved by the University of Navarra Ethics Committee on 22 December 2022 (ID: 2022.193 mod1; Chairperson: Dr María del Carmen Berasain Lasarte) and registered at ClinicalTrials.gov (NCT06925009). All procedures complied with the Declaration of Helsinki and Good Clinical Practice. Reporting followed TRIPOD recommendations for prediction model development studies, and STROBE recommendations where applicable.

### Participants

Consecutive adults (≥ 18 year) with American Society of Anesthesiologists Physical Status Classification System (ASA) physical status I–III scheduled for elective surgery requiring TI with VL (VL-TI) were enrolled between March 2023 and April 2025 at Hospital Universitario de Navarra (Madrid, Spain). Exclusion criteria were pregnancy, previous cervical radiotherapy, cervical tumours/goitre, cervical or maxillofacial malformations, and incomplete or invalid data. A total of 250 patients constituted the model-development cohort for the Airway Coach project.

### Pre-operative assessment

Demographics (age, sex, weight, height, BMI, ASA class) and standard airway predictors—modified Mallampati Score (MMS), thyromental distance (TMD), sternomental distance (SMD), inter-incisor distance (IID), upper-lip-bite test (ULBT) and neck circumference (NC)—were recorded in the anaesthesia preparation area by the principal investigator.

### Data collection and ultrasound measurements

Point-of-care ultrasound (POCUS) was performed in awake, neutrally positioned patients (cervical extension when necessary) using a LOGIQ V2 system (General Electric, Jiangsu, China). A high-frequency linear probe (6–12 MHz) assessed superficial, and a low-frequency convex probe (1–6 MHz) deep structures. All scans were done by a single anaesthesiologist with > 10 years’ dedicated airway-US experience.

**Fig. 1:**
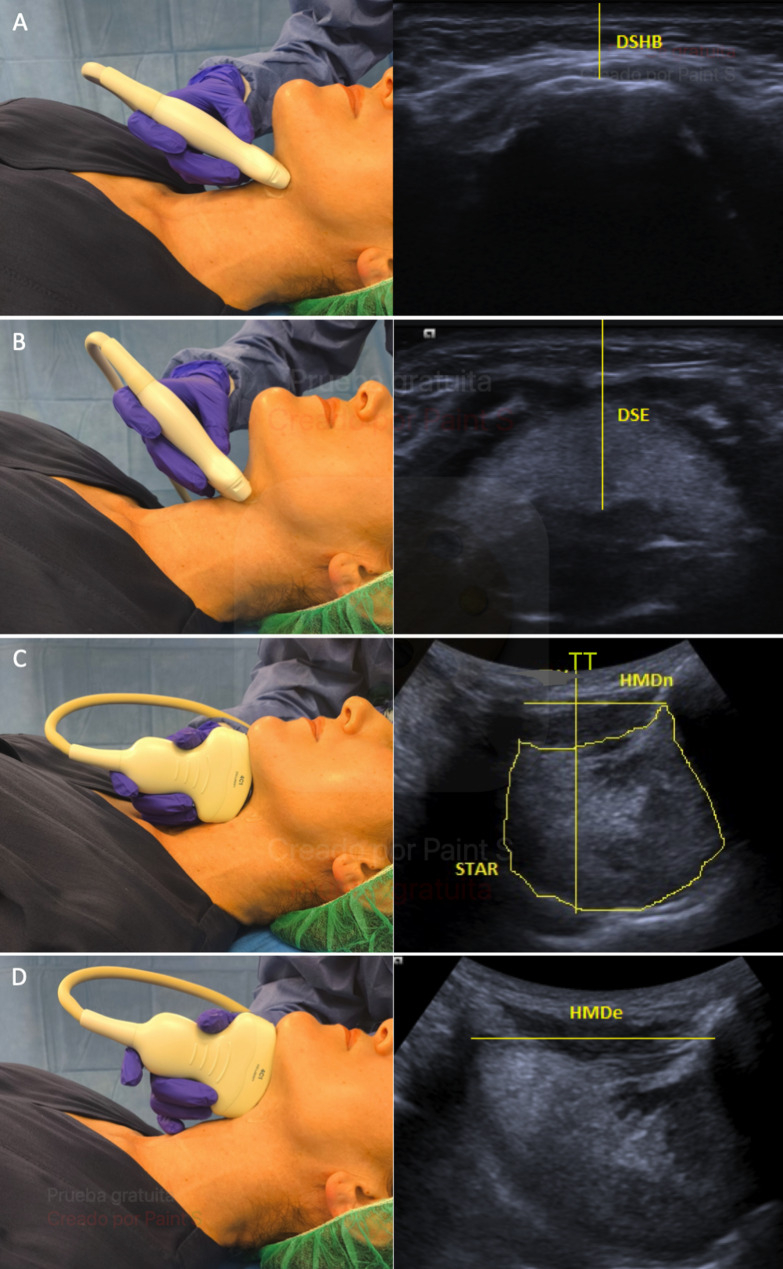
Airway ultrasound parameters used for machine learning-based prediction of videolaryngoscopy grade. Panel **A** Skin-to-hyoid bone distance (DSHB); Panel (**B**) Skin-to-epiglottis distance (DSE); Panel (**C**) Sagittal tongue area (STAR), tongue thickness (TT), and hyoid-to-mentum distance in neutral position (HMDn); Panel (**D**) Hyoid-to-mentum distance in extended position (HMDe). The hyoid-to-mentum ratio (HMDr) was calculated as HMDe divided by HMDn

Seven quantitative ultrasound variables were recorded: (Fig. 1)

1. Skin-to-hyoid distance (DSHB): distance from skin surface to hyoid bone. (Panel A)
2. Skin-to-epiglottis distance (DSE): distance from skin surface to epiglottis. (Panel B)
3. Hyoid-to-mentum distance, neutral (HMDn): distance from hyoid bone to mentum in neutral head position. (Panel C)
4. Hyoid-to-mentum distance, extended (HMDe): measured in maximal head extension. (Panel D)
5. Hyoid-to-mentum ratio (HMDr): calculated as HMDe/HMDn.
6. Sagittal tongue area (STAR): cross-sectional area of the tongue in sagittal view. (Panel C)
7. Tongue thickness (TT): distance from skin surface to deep palate. (Panel C)

### Anaesthesia and tracheal intubation

After preoxygenation using the tidal volume breathing technique until an end-tidal oxygen concentration (ETO₂) > 90% was achieved, anaesthesia was induced with propofol 2 mg kg⁻¹, fentanyl 1.5 µg kg⁻¹ and rocuronium 0.7 mg kg⁻¹. Face mask ventilation continued until complete neuromuscular block (train-of-four 0) and adequate hypnotic depth (BIS < 50). Standard monitoring comprised non-invasive blood pressure, ECG, pulse oximetry, capnography, neuromuscular and BIS monitoring. Tracheal intubation was initially performed with either a McGrath™ MAC (Medtronic, Minneapolis, MN, USA) or a C-MAC^®^ Macintosh blade (Karl-Storz, Tuttlingen, Germany), used as the initial devices. Blade size (3 or 4) was selected according to patient sex and anatomical features, in line with international guidelines and manufacturer recommendations. If the endotracheal tube could not be advanced toward the glottis, adjuncts—stylet, bougie or Frova airway intubation catheter (Cook Critical Care, Letchworth, UK)—were employed. In the event of inadequate glottic visualisation or failed tracheal intubation, a hyperangulated videolaryngoscope—Airtraq^®^ (Prodol Meditec, Vizcaya, Spain) or McGrath™ X3 blades (Medtronic, Minneapolis, MN, USA)—was used as a rescue device. Glottic view was quantified by the Percentage of Glottic Opening (POGO) score [17].

### Airway difficulty grading

Immediately after each videolaryngoscopic tracheal intubation, procedural difficulty—the study outcome—was prospectively classified using a functional grading system based on the Video Classification of Intubation (VCI) framework [18, 19]. The classification focused on intubation performance, distinguishing between scenarios in which glottic visualization was achieved but tracheal tube delivery differed in complexity [11] .

Procedural difficulty was categorized as follows:

- Grade 0: Successful first-pass tracheal intubation using a Macintosh-type videolaryngoscope without adjuncts.
- Grade 1: Adequate glottic visualization with the initial Macintosh-type videolaryngoscope, but tracheal intubation required adjuncts such as a stylet or tracheal tube introducer (*can see*,* can’t intubate easily*).
- Grade 2: Tracheal intubation could not be completed using the initial Macintosh-type videolaryngoscope despite adequate visualization and required escalation to a different device, typically a hyperangulated videolaryngoscope (*can see*,* can’t intubate with Macintosh blade*).

For machine-learning analysis, these categories were numerically encoded as Grades 0, 1, and 2.

Respiratory and haemodynamic variables were continuously monitored. Adverse events such as SpO₂ < 92%, oesophageal intubation, or dental trauma were recorded.

### Study objective

The primary aim was to develop a machine-learning model integrating clinical, demographic, and ultrasound variables to estimate, for each patient, the probability of classification into Grades 0–2, and to explore associations with videolaryngoscopy strategy and adjunct use.

### Statistical analysis and machine-learning pipeline

#### Sample size

This study was designed as a prospective exploratory model-development cohort. A sample size of 250 patients was considered appropriate for an initial proof-of-concept study, considering the expected distribution of videolaryngoscopy difficulty categories and the feasibility of prospective multimodal data collection, including airway ultrasound measurements [20].

#### Descriptive statistics 

Descriptive analyses were conducted using IBM SPSS Statistics v27.0 (IBM Corp., Armonk, NY, USA). Continuous variables are presented as mean ± standard deviation (SD) or median (interquartile range [IQR]), and categorical variables as counts (percentage).

#### Pre-processing 

Data completeness exceeded 99% for all predictors, and therefore no imputation was performed. Continuous predictors were standardised using a StandardScaler within the modelling pipeline to ensure comparable variable scales. Pre-processing steps were conducted in accordance with TRIPOD recommendations to support transparency and reproducibility; no resampling techniques (e.g. SMOTE) or synthetic data were used [21].

#### Predictor selection 

The dataset was randomly split into training (80%) and test (20%) sets. Embedded variable selection was performed using a Random Forest (RF) approach within the training dataset, with feature selection integrated into the cross-validation pipeline to avoid data leakage. The selected predictors were subsequently used for model development across all algorithms. Stratified k-fold cross-validation (k = 10) was applied within the training set, and final model performance was evaluated on the independent test set [22].

#### Model selection 

Four algorithms were selected for their complementarity in multiclass classification tasks and relative interpretability in a clinical research context [23]:

- a. Random Forest (RF): ensemble model robust to multivariate and non-linear data, providing measures of variable importance.
- b. Support Vector Machine (SVM): linear kernel, suitable for high-dimensional data, implemented using a one-vs-rest strategy for multiclass classification.
- c. Extreme Gradient Boosting (XGBoost): gradient boosting algorithm efficient for heterogeneous clinical datasets.
- d. Multinomial logistic regression: classical linear model used as a reference comparator, implemented in multinomial mode with a one-vs-rest strategy.

#### Training, optimisation, and validation

Models were trained using stratified k-fold cross-validation (k = 10) within the training dataset. Hyperparameters were optimised using grid search within the cross-validation framework. Hyperparameter optimisation was performed using GridSearchCV with stratified 10-fold cross-validation (StratifiedKFold, shuffle = True, random_state = 42), and model selection was based on macro-averaged one-vs-rest ROC-AUC (roc_auc_ovr). Additional details regarding hyperparameter search grids and selected configurations are provided in Supplementary Table 2. Final model performance was evaluated on the independent test set using macro-averaged area under the receiver operating characteristic curve (AUC-ROC), accuracy, sensitivity, specificity, and F1-score, accounting for the multiclass nature of the outcome. Macro-averaged metrics were used to mitigate the influence of class imbalance and to ensure balanced performance assessment across outcome categories.

Model development and internal evaluation were conducted using Python (version 3.11.8, Anaconda distribution) with the following libraries: pandas 2.2.0, numpy 1.26.4, scikit-learn 1.5.0, xgboost 2.0.3, and matplotlib 3.9.0.

## Results

### Study population

Of 250 enrolled patients, 187 (74.8%) were intubated on the first videolaryngoscopic attempt using a Macintosh blade (Grade 0), 44 (17.6%) required adjuncts such as a stylet or Frova^®^ catheter (Grade 1), and 19 (7.6%) required escalation to a hyperangulated videolaryngoscope (Grade 2). Baseline demographic, clinical, and ultrasound characteristics by grade are summarised in Table 1.

**Table 1:** Demographic, clinical, and ultrasonographic characteristics stratified by airway management grade (Airway coach classification)

Variable	Grade 0 (*n* = 187)	Grade 1 (*n* = 44)	Grade 2 (*n* = 19)
*Demographic*			
Age (years)	56.9 (15.7)	59.6 (13.1)	64.2 (10.0)
BMI (kg/m²)	25.7 (4.4)	28.6 (3.7)	27.8 (4.0)
Sex (M/F)	86 (46.0%) / 101 (54.0%)	12 (27.3%) / 32 (72.7%)	5 (26.3%) / 14 (73.7%)
ASA I–II / III	174 (93.0%) / 13 (7.0%)	39 (88.6%) / 5 (11.4%)	15 (78.9%) / 4 (21.1%)
*Clinical*			
MMS I–II / III–IV	170 (90.9%) / 17 (9.1%)	32 (72.7%) / 12 (27.3%)	12 (63.2%) / 7 (36.8%)
TMD (cm)	7.6 (0.8)	7.4 (0.8)	7.0 (1.2)
SMD (cm)	13.9 (1.5)	13.5 (1.4)	13.0 (1.7)
ID (cm)	4.3 (0.6)	4.1 (0.9)	4.1 (0.6)
ULBT I–II / III	3 (1.6%) / 184 (98.4%)	2 (4.5%) / 42 (95.5%)	2 (10.5%) / 17 (89.5%)
NC (cm)	38.3 (3.7)	42.0 (3.7)	42.1 (3.2)
*Ultrasound*			
DSHB (cm)	1.00 (0.22)	1.33 (0.28)	1.48 (0.22)
DSE (cm)	1.94 (0.34)	2.53 (0.33)	2.68 (0.16)
HMDn (cm)	4.81 (0.55)	5.13 (0.61)	4.98 (0.68)
HMDe (cm)	5.57 (0.61)	5.74 (0.73)	5.61 (0.71)
HMD ratio	1.16 (0.09)	1.11 (0.06)	1.17 (0.13)
TT (cm)	5.59 (0.61)	6.26 (0.66)	5.97 (0.76)
STAR (cm²)	20.1 (2.5)	26.6 (3.0)	26.6 (2.0)

Data are expressed as mean (SD) for continuous variables and as number (percentage) for categorical variables

*Abbreviations*: *BMI* Body Mass Index, *ASA* American Society of Anesthesiologists (classification), *MMS* Modified Mallampati Score, *TMD* Thyromental Distance, *SMD* Sternomental Distance, *ID* Interincisor Distance, *ULBT* Upper Lip Bite Test, *NC* Neck Circumference, *DSHB* Distance from Skin to Hyoid Bone, *DSE* Distance from Skin to Epiglottis, *HMDn* Hyomental Distance in neutral position, *HMDe* Hyomental Distance in extended position, *HMD ratio* Hyomental Distance Ratio (HMDe/HMDn), *TT* Tongue Thickness, *STAR* Sagittal Tongue Area

### Machine learning analysis

#### Feature selection

Random Forest (RF) embedded feature selection identified six predictors—sagittal tongue area (STAR), skin-to-epiglottis distance (DSE), skin-to-hyoid distance (DSHB), tongue thickness (TT), body-mass index (BMI), and age—which were retained for model development across all algorithms (Table 2). 

**Table 2:** Relative importance of predictive variables in the Random Forest model. Variable importance reflects each feature’s contribution to node impurity reduction within the model. Normalised importance is expressed as a percentage relative to the most influential variable (STAR)

Rank	Feature (unit)	Relative Importance	Normalised Importance (%)	Direction of Association*
1	Sagittal tongue area (STAR, cm²)	0.29	100.0	Larger → ↑ difficulty
2	Skin-to-epiglottis distance (DSE, cm)	0.27	98.8	Longer → ↑ difficulty
3	Tongue thickness (TT, cm)	0.13	47	Thicker → ↑ difficulty
4	Skin-to-hyoid distance (DSHB, cm)	0.10	36.2	Longer → ↑ difficulty
5	Body-mass index (BMI, kg m⁻²)	0.08	28.6	Higher → ↑ difficulty
6	Age (yr)	0.07	25.8	Older → ↑ difficulty

*Direction of association indicates the general relationship between each variable and increasing procedural difficulty, as derived from the model. The upward arrow (↑) denotes an association with higher predicted difficulty. These directions reflect overall trends and should not be interpreted as causal effects

#### Model performance

Model performance metrics derived from stratified 10-fold cross-validation and from the independent test set (*n* = 50) are reported in Table 3. In the independent test set, Random Forest and Extreme Gradient Boosting (XGBoost) achieved the highest overall accuracy (92%), outperforming support vector machine and multinomial logistic regression. Performance differed across outcome categories, with greater variability in F1-scores for Grades 1 and 2.

**Table 3:** Performance metrics of machine learning models for predicting videolaryngoscopy grade (Airway Coach classification)

Model	Accuracy	AUC (CV ± SD)	95% CI	Macro F1	Grade 0 F1	Grade 1 F1	Grade 2 F1
Logistic Regression	0.88	0.92 ± 0.04	[0.891–0.949]	0.66	0.97	0.67	0.33
Support Vector Machine	0.88	0.93 ± 0.05	[0.894–0.966]	0.56	0.97	0.70	0.00
Random Forest	0.92	0.95 ± 0.02	[0.936–0.964]	0.76	1.00	0.78	0.50
XGBoost	0.92	0.95 ± 0.03	[0.928–0.972]	0.80	0.99	0.75	0.67

*AUC* area under the receiver operating characteristic curve (cross-validated), *Macro F1* unweighted mean of class-specific F1 scores, *Grade-specific F1* harmonic mean of precision and recall for each grade

Both XGBoost and Random Forest achieved identical overall accuracy (92%) and mean AUC (0.95). However, XGBoost demonstrated superior class-wise performance in the most challenging category (Grade 2, F1-score = 0.67), indicating a more balanced discriminative capacity and supporting its selection as the optimal model for personalised airway management prediction

Macro-F1 scores and precision–recall analyses are reported in Supplementary Tables 1 and Supplementary Fig. 1 to further contextualise model performance in the presence of class imbalance.

#### Discrimination

**Fig. 2:**
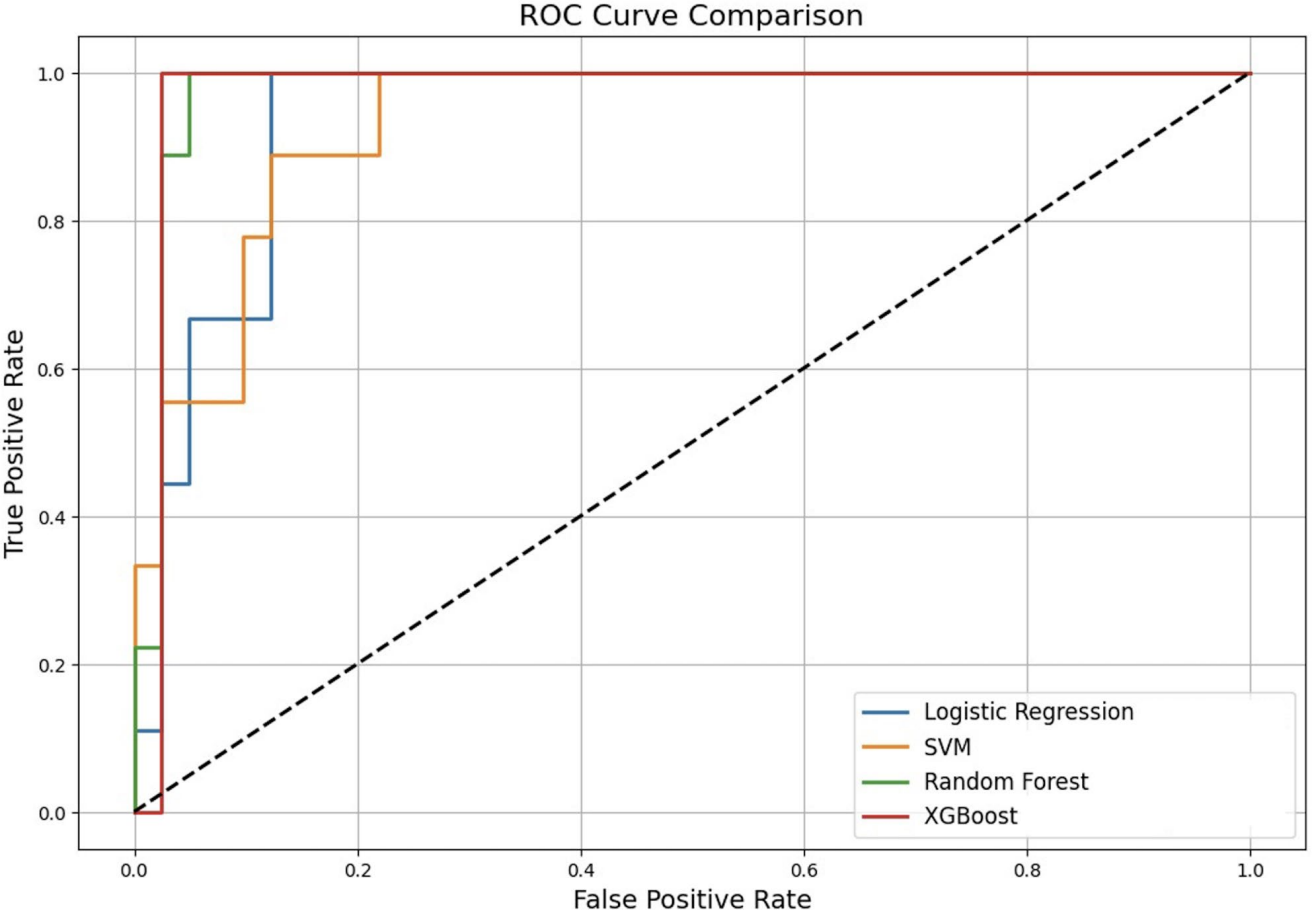
Receiver-operating-characteristic (ROC) curves comparing model performance on the independent test set. Fig. 2. Receiver operating characteristic (ROC) curves comparing the discriminative performance of four machine-learning models for predicting videolaryngoscopy-related procedural difficulty (Grades 0–2, Airway Coach classification) in the independent test set. Random Forest and Extreme Gradient Boosting (XGBoost) demonstrated similarly high discrimination (AUC ≈ 0.95), exceeding that of support vector machine (AUC ≈ 0.93) and multinomial logistic regression (AUC ≈ 0.92). ROC curves are shown within the test cohort

Receiver operating characteristic (ROC) curves in the independent test set demonstrated differences in discriminative performance among models for classifying videolaryngoscopy-related procedural difficulty. Random Forest (AUC = 0.95 ± 0.02; 95% CI: 0.936–0.964) and Extreme Gradient Boosting (XGBoost) (AUC = 0.95 ± 0.03; 95% CI: 0.928–0.972) showed higher AUC values than Support Vector Machine (AUC = 0.93 ± 0.05; 95% CI: 0.894–0.966) and multinomial logistic regression (AUC = 0.92 ± 0.04; 95% CI: 0.891–0.949) (Fig. 2; Table 3).

#### Confusion matrix

**Fig. 3:**
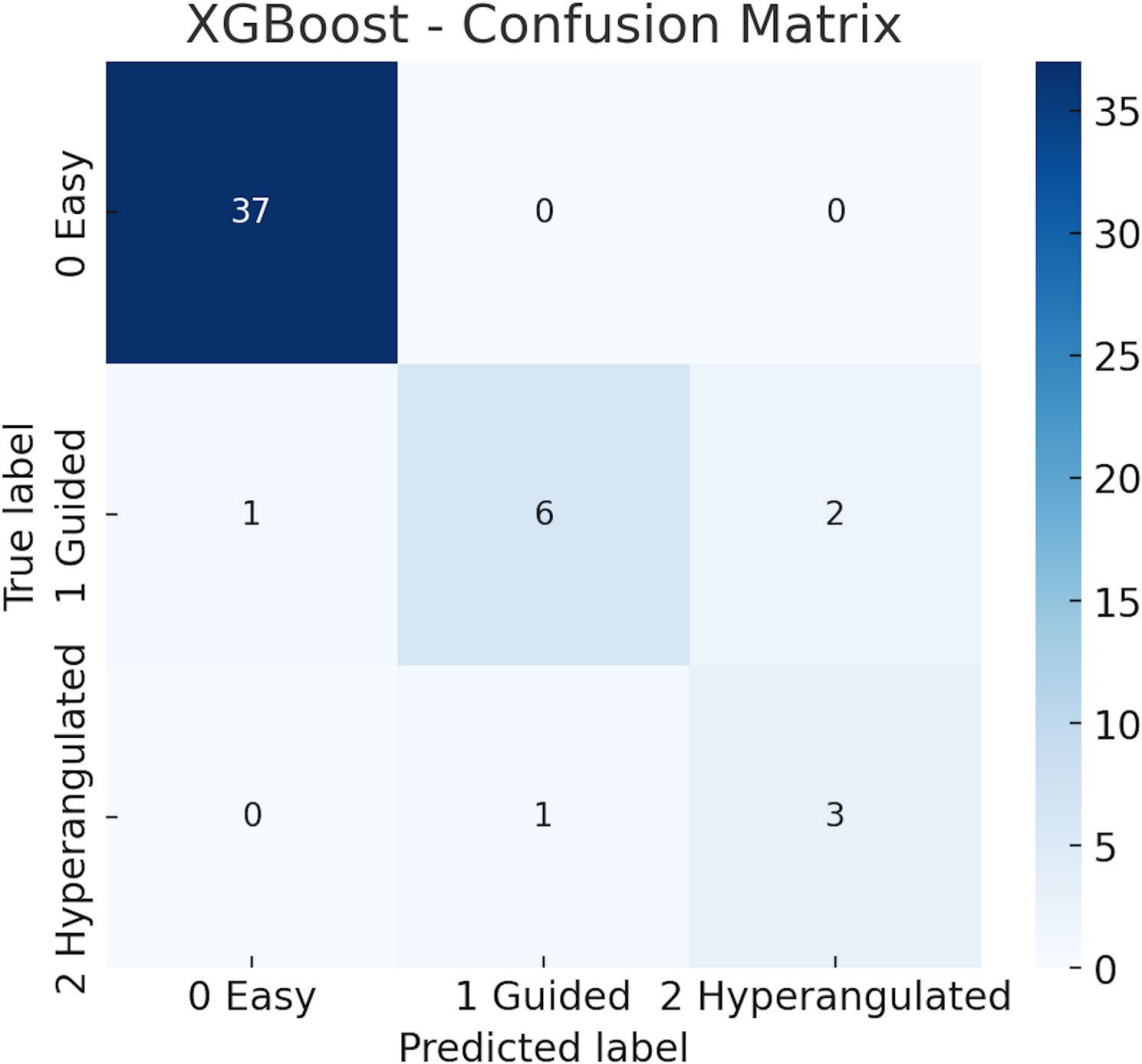
Confusion matrix of the XGBoost model for videolaryngoscopy grade prediction (Airway Coach classification). The matrix compares the true labels (rows) versus the predicted labels (columns) for three videolaryngoscopy strategies: Grade 0 (easy intubation), Grade 1 (requiring a stylet or guide), and Grade 2 (requiring a hyperangulated blade)

The XGBoost confusion matrix (Fig. 3) demonstrated correct classification of all Grade 0 cases, with no misclassification of higher-difficulty airways as Grade 0. Classification performance for Grade 1 and Grade 2 was more variable, with sensitivities of 66% and 75% and specificities of 97% and 96%, respectively. Misclassification of higher-difficulty airways as Grade 0 was uncommon, with only one Grade 1 case incorrectly classified as Grade 0.

Across all evaluated models, Random Forest and XGBoost demonstrated comparable overall discriminative performance. However, XGBoost showed a more balanced class-wise performance, particularly for the highest-difficulty category (Grade 2).

### Calibration analysis

Calibration analyses, including Brier scores and reliability diagrams, are reported in Supplementary Tables 3 and Supplementary Fig. 2. XGBoost demonstrated the lowest macro-Brier score, suggesting generally good agreement between predicted probabilities and observed outcomes. Calibration estimates for minority outcome categories showed greater variability, particularly for Grade 2, reflecting the limited number of observations in the independent test set.

## Discussion

In this single-centre cohort of adults undergoing elective surgery, we developed and internally evaluated a multimodal machine-learning model integrating clinical characteristics and airway ultrasound variables to classify videolaryngoscopy-related procedural difficulty. The model was anchored to a functional, videolaryngoscopy-specific outcome that captures scenarios in which adequate or optimal glottic visualisation does not necessarily translate into straightforward tracheal tube delivery—the so-called “can see, can’t intubate” phenomenon [11]. By focusing on the need for adjuncts or escalation to an alternative videolaryngoscope despite satisfactory visualisation, this framework reflects contemporary videolaryngoscopy practice and provides a structured approach to pre-procedural airway planning.

Pre-procedural airway evaluation is universally recommended and remains a cornerstone of safe anaesthetic practice [6, 8]. Its primary value lies in enabling structured planning, not only through the selection of an appropriate first-line airway strategy but also by anticipating escalation pathways should tracheal intubation prove difficult. Such risk stratification supports rational resource allocation, facilitates timely transitions between airway plans, and reinforces cognitive preparedness for unanticipated difficulty. Despite this central role, the diagnostic accuracy of classical bedside airway assessment tests remains limited [3]. Most commonly used univariate predictors—such as the modified Mallampati score, thyromental distance, and the upper lip bite test—were originally developed to predict difficult direct laryngoscopy rather than videolaryngoscopy and consistently demonstrate low sensitivity and poor negative predictive value for difficult tracheal intubation [24]. For example, while the upper lip bite test has been reported to offer the highest sensitivity for difficult direct laryngoscopy, its performance remains modest, and for difficult tracheal intubation the modified Mallampati score performs only marginally better than chance [25].

Multivariable airway assessment tools represent an evolution toward more structured and multifactorial risk stratification. Scores such as the Wilson score and the MACOCHA index incorporate combinations of anatomical, physiological, and contextual variables and may offer improved discrimination compared with single-parameter tests [26]. However, these tools remain under-investigated in the context of videolaryngoscopy, are often derived from heterogeneous populations, and frequently lack robust external validation. The MACOCHA score—the only tool validated in critically ill patients—combines patient-related, pathological, and operator-dependent factors, yet still fails to reliably identify a substantial proportion of difficult airways [27]. Therefore, most difficult tracheal intubations remain unanticipated, accounting for a large proportion of unexpected intubations and airway-related adverse events [28]. Although the widespread adoption of videolaryngoscopy has improved glottic visualisation and first-pass success, adverse outcomes may still occur when blade geometry or adjunct use is not adequately matched to patient-specific anatomy. These limitations underscore the need for approaches that move beyond traditional clinical scores and integrate additional sources of anatomical information to better characterise videolaryngoscopy-related procedural difficulty.

These considerations are particularly relevant in the context of videolaryngoscopy, where excellent or even optimal glottic visualisation does not necessarily translate into straightforward tracheal tube delivery. The well-recognised “can see, can’t intubate” or “can see, can’t intubate easily” scenarios highlight the importance of factors beyond visualisation alone, including tube trajectory, blade geometry, and the timely use of adjuncts [11]. Contemporary airway management guidelines emphasise the critical importance of optimising the first intubation attempt and avoiding repeated, poorly planned attempts, advocating instead for a structured, stepwise escalation strategy when difficulty is encountered [10].

From this perspective, anticipating whether a standard Macintosh-type videolaryngoscope is likely to be sufficient, or whether adjuncts or an alternative blade geometry may be required, aligns closely with guideline-based principles of airway safety [10]. While tracheal tube introducers and stylets remain essential tools in modern airway management, their use—particularly when unanticipated or repeated—has been associated with airway trauma in certain clinical contexts [13]. Anticipating the need for adjuncts or early escalation to a different videolaryngoscope may therefore contribute to a more controlled and deliberate intubation strategy, consistent with recommendations to prioritise first-pass success and minimise airway injury.

Point-of-care airway ultrasound is now recognised as a powerful adjunct to bedside airway assessment. Ultrasound-derived metrics such as the skin-to-epiglottis (DSE) and skin-to-hyoid (DSHB) distances have consistently outperformed classical clinical tests in predicting airway difficulty, particularly by capturing soft-tissue anatomy that is not reflected by external examination alone [29]. Multimodal clinical–ultrasound scores have begun to emerge; however, most were developed in the context of direct laryngoscopy, despite videolaryngoscopy now being recommended as a first-line technique in multiple international guidelines [30].

A recent pilot study combined ultrasound variables with predictors from the Canadian Airway Focus Group to estimate videolaryngoscopy difficulty [31]. To date, no widely validated model has integrated ultrasound, clinical, and demographic data within an artificial intelligence–based framework specifically designed to anticipate videolaryngoscopy-related procedural challenges, including blade selection and adjunct use. In this context, Airway Coach explores a multimodal approach by combining six anatomically and clinically plausible predictors within a machine-learning framework, laying the groundwork for future data-driven strategies to support personalised videolaryngoscopy planning.

The model achieved an area under the receiver operating characteristic curve of 0.95 and an overall accuracy of 92% on the independent test set, with higher discrimination for the most complex videolaryngoscopy category (Grade 2). These results indicate that a multimodal machine-learning approach can capture clinically relevant patterns associated with increased videolaryngoscopy-related procedural difficulty.

Given the marked class imbalance within the cohort, macro-averaged F1-scores and precision–recall analyses were additionally evaluated to better contextualise model performance across outcome categories. These analyses demonstrated lower performance for the least represented category (Grade 2), highlighting the challenges associated with minority-class prediction in exploratory multiclass machine-learning models developed from moderate-sized cohorts.

Recent studies have explored the application of machine learning models to predict difficult laryngoscopy, demonstrating improved performance compared to conventional regression approaches. For example, a large emergency department cohort showed that ensemble methods such as Random Forest can achieve higher discriminative performance than traditional statistical models [32]. However, these approaches remain fundamentally anchored to the prediction of laryngoscopic view, typically defined by Cormack–Lehane grading.

When benchmarked against recently published artificial intelligence–based airway assessment models relying on facial images, cervical radiographs, or generic deep-learning pipelines, these results are consistent with the growing evidence that machine-learning and computer-vision approaches can outperform traditional bedside airway assessment tools [33–37]. Nevertheless, most existing machine learning models—whether based on clinical variables, videolaryngoscopy-derived features [38], or deep learning approaches applied to airway imaging [39]—continue to focus on anatomical surrogates such as laryngoscopic view, which may not adequately reflect procedural complexity or intubation success [40]. In contrast, the present model uses a videolaryngoscopy-specific endpoint that captures not only glottic visualisation but also intubation performance, including the need for adjuncts or escalation to a hyperangulated blade. This distinction is particularly relevant in videolaryngoscopy, where an excellent laryngeal view may coexist with difficulty advancing the tracheal tube—the well-recognised “can see, can’t intubate” scenario—and where higher Cormack–Lehane grades are infrequently encountered, further limiting the utility of that scale [31].

This study has several limitations that merit consideration. First, it was conducted at a single centre with a moderate sample size (*n* = 250) and a relatively small proportion of higher-difficulty cases (Grades 1–2), which may limit statistical power and increase the risk of overfitting despite the use of cross-validation strategies. In particular, the combination of a limited sample size and a multiclass outcome may affect model stability and reduce the reliability of class-specific performance estimates. The marked class imbalance, with approximately 75% of cases classified as Grade 0, may also have influenced performance metrics, favouring easier airways. To mitigate these limitations, we used stratified cross-validation, an independent test set, and macro-averaged performance metrics; however, the results should be interpreted with caution, especially for the less frequent classes.

All ultrasound measurements were obtained by a single experienced operator, and therefore inter-operator reproducibility across different levels of ultrasound expertise was not assessed. This may limit the generalisability of the findings, as measurement variability could differ in routine clinical practice. However, airway ultrasound is an increasingly standardised technique, and previous studies have demonstrated acceptable inter-operator reproducibility after appropriate training. Future studies should evaluate model performance in settings with operators of varying levels of experience to better assess its feasibility and scalability in real-world practice. In addition, device generalisability remains uncertain, as only two Macintosh-type videolaryngoscopes were evaluated. The study population was limited to elective surgical patients with ASA physical status I–III, excluding emergency, obstetric, paediatric, and critically ill populations, and only static pre-operative variables were analysed, without accounting for dynamic intra-operative factors that may influence intubation performance.

The videolaryngoscopy difficulty outcome was based on a pragmatic 0–1–2 classification aligned with the Video Classification of Intubation framework, which has not yet been externally validated in this specific configuration. This three-category structure reflected the stepwise videolaryngoscopy strategy used in our centre while maintaining sufficient representation within each outcome group for exploratory multiclass model development. A more granular classification could potentially identify additional procedural subgroups, particularly in cases requiring advanced rescue strategies despite hyperangulated videolaryngoscopy; however, such scenarios were extremely infrequent in the present cohort and would likely have resulted in very small or empty categories. Furthermore, although XGBoost demonstrated strong predictive performance, its limited interpretability compared with simpler statistical models may constrain direct clinical adoption without additional explainability strategies. Finally, this study did not address feasibility aspects such as cost, workflow integration, or environmental impact, as model development and evaluation were performed offline.

## Conclusion

This study demonstrates the feasibility of a machine learning–based approach to support personalised videolaryngoscopy strategy planning in a single-centre observational cohort. By integrating readily available clinical, demographic, and airway ultrasound variables, the proposed model showed robust discriminative performance for anticipating videolaryngoscopy-related procedural difficulty, including the need for adjuncts or escalation in blade geometry.

These findings support the role of data-driven methods as a complement to conventional airway assessment, with the potential to enhance pre-procedural planning and decision-making in videolaryngoscopy. Further evaluation in independent populations is required to assess generalisability, calibration, and clinical impact before implementation in routine practice.

## Supplementary Information

Supplementary Material 1.

Supplementary Material 2.

Supplementary Material 3.

Supplementary Material 4.

Supplementary Material 5.

## Data Availability

The datasets generated and/or analysed during the current study are not publicly available due to institutional data protection policies but are available from the corresponding author on reasonable request.

## Abbreviations

ASA: American Society of Anesthesiologists
AUC: Area under the receiver operating characteristic curve
BMI: Body mass index
BIS: Bispectral index
CI: Confidence interval
DL: Direct laryngoscopy
DSE: Skin–to–epiglottis distance
DSHB: Skin–to–hyoid distance
ETO₂: End–tidal oxygen concentration
HMDe: Hyoid–to–mentum distance in head extension
HMDn: Hyoid–to–mentum distance in neutral position
HMDr: Hyoid–to–mentum ratio
IID: Inter–incisor distance
IQR: Interquartile range
ML: Machine learning
MMS: Modified Mallampati score
NC: Neck circumference
POCUS: Point–of–care ultrasound
POGO: Percentage of glottic opening
RF: Random Forest
ROC: Receiver operating characteristic
SD: Standard deviation
SMD: Sternomental distance
STAR: Sagittal tongue area
SVM: Support Vector Machine
TI: Tracheal intubation
TMD: Thyromental distance
TT: Tongue thickness
ULBT: Upper lip bite test
VCI: Video Classification of Intubation
VL: Videolaryngoscopy
XGBoost: Extreme Gradient Boosting
