# Supplementary material for: Airway coach project: development of a machine learning–based model using clinical and ultrasound parameters to support videolaryngoscopy strategy
Source: BMC Anesthesiol. 2026 Jun 18;26:456. doi: 10.1186/s12871-026-03943-4 (PMC13425934; doi:10.1186/s12871-026-03943-4)
Supplement: Supplementary file 1 — Supplementary Material 1. [file 12871_2026_3943_MOESM1_ESM.docx]

**Supplementary Table 1**

**Precision–recall performance metrics across machine-learning models and videolaryngoscopy difficulty categories**

**Panel A.** Precision–recall area under the curve (PR-AUC; Average Precision) obtained from stratified 10-fold cross-validation.

| **Model** | **Grade 0** | **Grade 1** | **Grade 2** | **Macro PR-AUC** |
| --- | --- | --- | --- | --- |
| XGBoost | 0.969 | 0.674 | 0.556 | 0.733 |
| Random Forest | 0.986 | 0.696 | 0.510 | 0.730 |
| Logistic Regression | 0.982 | 0.636 | 0.426 | 0.681 |
| SVM | 0.979 | 0.573 | 0.351 | 0.634 |

**Panel B.** Precision–recall area under the curve (PR-AUC; Average Precision) obtained from the independent test set (n = 50).

| **Model** | **Grade 0** | **Grade 1** | **Grade 2** | **Macro PR-AUC** |
| --- | --- | --- | --- | --- |
| XGBoost | 1.000 | 0.832 | 0.778 | 0.870 |
| Random Forest | 1.000 | 0.873 | 0.725 | 0.866 |
| Logistic Regression | 0.996 | 0.790 | 0.591 | 0.792 |
| SVM | 0.996 | 0.776 | 0.549 | 0.774 |

PR-AUC = area under the precision–recall curve (Average Precision). Macro PR-AUC values correspond to the unweighted mean across outcome categories. Lower PR-AUC values in minority classes reflect the limited representation of higher-difficulty videolaryngoscopy categories within the cohort.
