# Supplementary material for: Airway coach project: development of a machine learning–based model using clinical and ultrasound parameters to support videolaryngoscopy strategy
Source: BMC Anesthesiol. 2026 Jun 18;26:456. doi: 10.1186/s12871-026-03943-4 (PMC13425934; doi:10.1186/s12871-026-03943-4)
Supplement: Supplementary file 2 — Supplementary Material 2. [file 12871_2026_3943_MOESM2_ESM.docx]

**Supplementary Table 2.**

**Hyperparameter search grids and selected model configurations**

| Model | Hyperparameter | Search space | Selected |
| --- | --- | --- | --- |
| Logistic Regression | C | {0.01, 0.1, 1, 10, 100} | 0.1 |
| Penalty | {L1, L2} | L2 |  |
| Solver | {saga} | saga |  |
| Configurations evaluated | 10 | — |  |
| Support Vector Machine (SVM) | C | {0.1, 1, 10} | 0.1 |
| Kernel | {linear, RBF} | linear |  |
| Gamma | {0.01, 0.1, 1} | 0.01 |  |
| Configurations evaluated | 18 | — |  |
| Random Forest | n_estimators | {100, 200, 300} | 100 |
| max_depth | {None, 4, 8} | None |  |
| max_features | {sqrt(p), log₂(p)} | sqrt(p) |  |
| Criterion | {gini, entropy} | entropy |  |
| Configurations evaluated | 36 | — |  |
| Extreme Gradient Boosting (XGBoost) | n_estimators | {100, 200, 300} | 100 |
| max_depth | {3, 4, 5} | 3 |  |
| learning_rate | {0.01, 0.1, 0.2} | 0.2 |  |
| subsample | {0.8, 1.0} | 0.8 |  |
| colsample_bytree | {0.8, 1.0} | 0.8 |  |
| gamma | {0, 0.1, 0.2} | 0.1 |  |
| Configurations evaluated | 324 | — |  |

Hyperparameter optimisation was performed using GridSearchCV with stratified 10-fold cross-validation (StratifiedKFold, shuffle = True, random_state = 42). Model selection was based on macro-averaged one-vs-rest ROC-AUC (roc_auc_ovr). Continuous predictors were standardised within the training partition to minimise data leakage.
