# Supplementary material for: Airway coach project: development of a machine learning–based model using clinical and ultrasound parameters to support videolaryngoscopy strategy
Source: BMC Anesthesiol. 2026 Jun 18;26:456. doi: 10.1186/s12871-026-03943-4 (PMC13425934; doi:10.1186/s12871-026-03943-4)
Supplement: Supplementary file 3 — Supplementary Material 3. [file 12871_2026_3943_MOESM3_ESM.docx]

**Supplementary Table 3.**

**Brier scores on the independent test set (one-vs-rest, per class)**

| **Model** | **Grade 0 (Easy)** | **Grade 1 (Difficult)** | **Grade 2 (Failed)** | **Macro Brier** |
| --- | --- | --- | --- | --- |
| **XGBoost** | 0.002 | 0.047 | 0.045 | 0.031 |
| **Random Forest** | 0.013 | 0.062 | 0.052 | 0.042 |
| **Support Vector Machine (SVM)** | 0.033 | 0.079 | 0.054 | 0.055 |
| **Logistic Regression** | 0.043 | 0.082 | 0.051 | 0.059 |

Lower Brier scores indicate better probabilistic calibration and overall predictive accuracy. Macro-Brier values correspond to the unweighted mean across outcome categories. Calibration estimates for minority outcome categories should be interpreted cautiously given the limited number of Grade 2 cases in the independent test set.
