# Supplementary figures and images for: Airway coach project: development of a machine learning–based model using clinical and ultrasound parameters to support videolaryngoscopy strategy
Source: BMC Anesthesiol. 2026 Jun 18;26:456. doi: 10.1186/s12871-026-03943-4 (PMC13425934; doi:10.1186/s12871-026-03943-4)

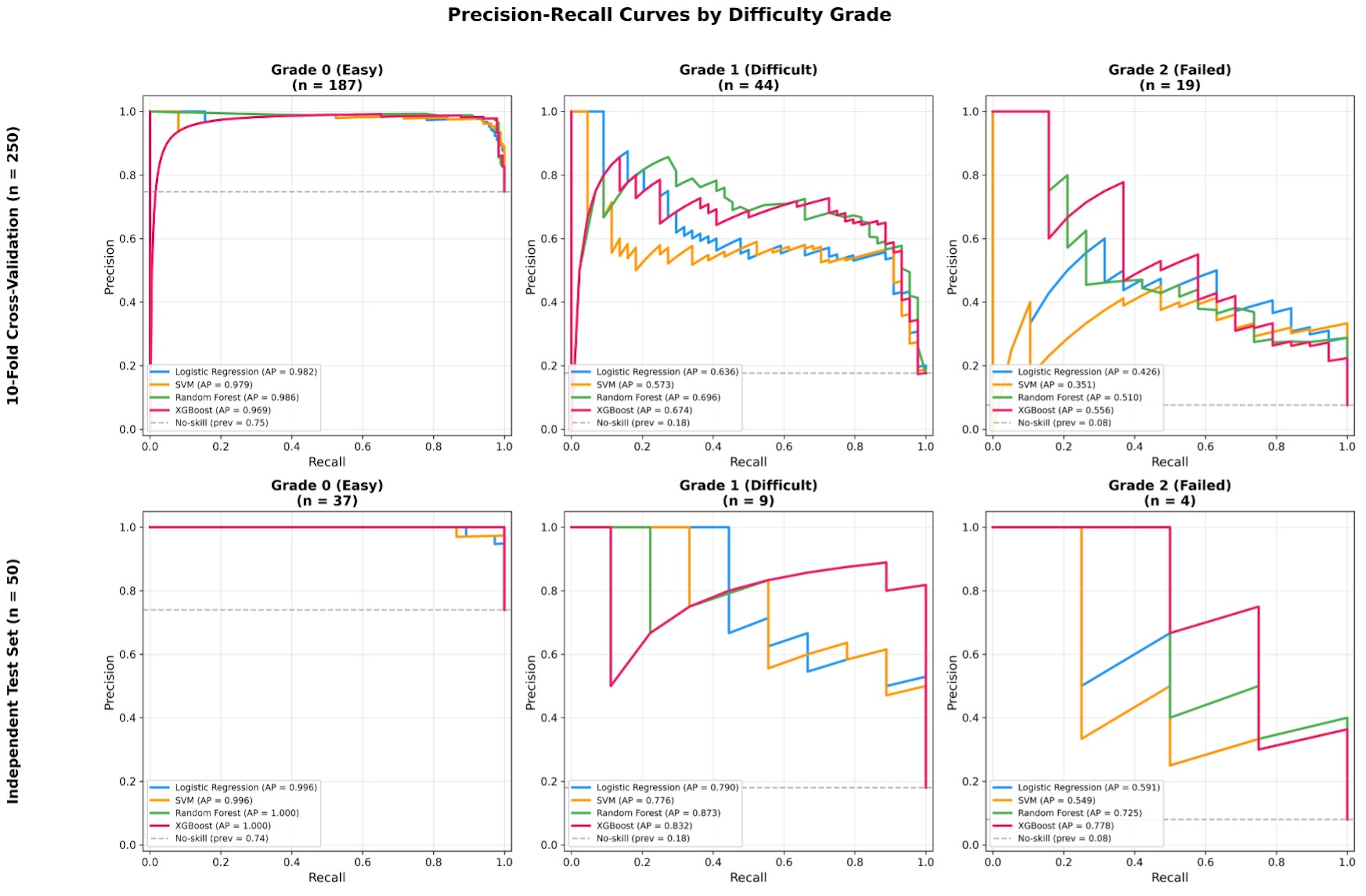

Supplement: Supplementary file 4 — Supplementary Material 4. [file 12871_2026_3943_MOESM4_ESM.jpg]

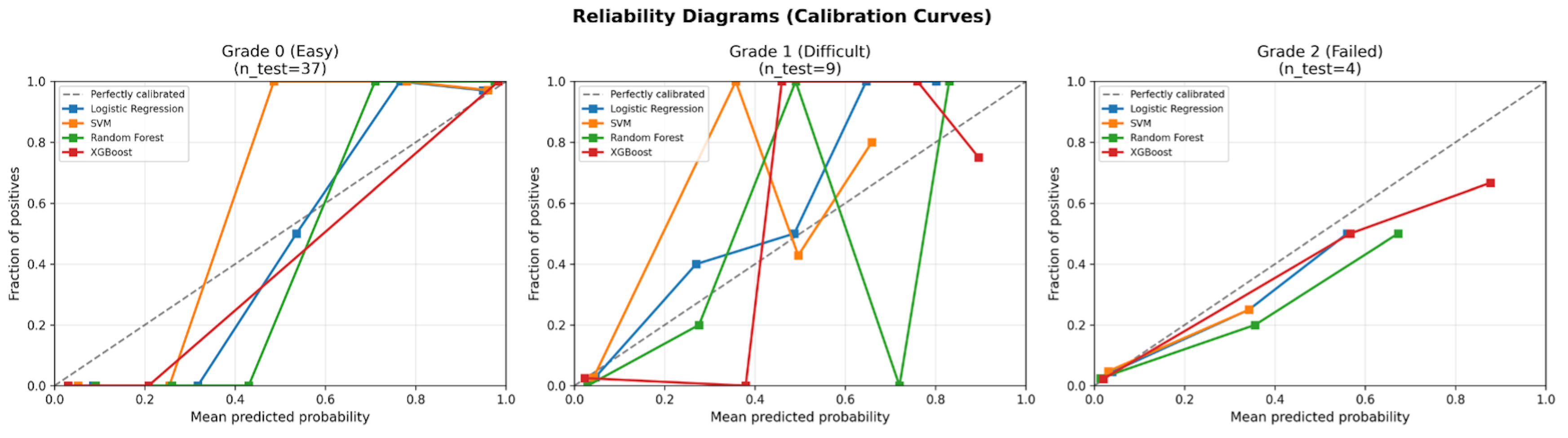

Supplement: Supplementary file 5 — Supplementary Material 5. [file 12871_2026_3943_MOESM5_ESM.png]
